# Polymer Electrolytes for Lithium/Sulfur Batteries

**DOI:** 10.3390/membranes2030553

**Published:** 2012-08-09

**Authors:** Yan Zhao, Yongguang Zhang, Denise Gosselink, The Nam Long Doan, Mikhail Sadhu, Ho-Jae Cheang, Pu Chen

**Affiliations:** Department of Chemical Engineering, University of Waterloo, 200 University Avenue West, Waterloo, Ontario N2L3G1, Canada; Email: y236zhao@uwaterloo.ca (Y.Z.); y378zhan@uwaterloo.ca (Y.Z.); denise@uwaterloo.ca (D.G.); doannamlong@yahoo.com (T.N.L.D.); mikhailsadhu@hotmail.com (M.S.); hcheang@uwaterloo.ca (H.-J.C.)

**Keywords:** polymer electrolyte, lithium-sulfur battery, solid polymer electrolyte, gel polymer electrolyte

## Abstract

This review evaluates the characteristics and advantages of employing polymer electrolytes in lithium/sulfur (Li/S) batteries. The main highlights of this study constitute detailed information on the advanced developments for solid polymer electrolytes and gel polymer electrolytes, used in the lithium/sulfur battery. This includes an in-depth analysis conducted on the preparation and electrochemical characteristics of the Li/S batteries based on these polymer electrolytes.

## 1. Introduction

With the rapid exhaustion of limited resources such as fossil fuels and their global environmental issues, the modern society’s sustainability depends on the development of ecological alternative power sources such as solar and wind energy, and low-emission transportation such as hybrid and electric vehicles. High energy-density batteries are an essential part of such systems and there is an insatiable demand for its improvement. Secondary lithium-ion batteries (LIBs) dominate the market for portable electronics (e.g., cellular phones, notebook computers, camcorders) [1,2,3], but are still not economically attractive for large scale and transportation applications. 

Although tremendous progress has been achieved in the field of LIBs, the transition metal oxides and phosphates typically used as cathode materials have maximum theoretical capacity limited to about 200 mAh·g^−1^ (of which only 170 mAh·g^−1^ can be practically achieved) [4,5,6,7]. The energy limitations, along with the high cost and ecological concerns of these materials, can restrict their practical application in large scale scenarios.

An alternative technology under intense development is the lithium/sulfur battery (Li/S). Elemental sulfur has higher theoretical capacity (1672 mAh·g^−1^) and specific energy (2600 Wh·kg^−1^) than conventional cathode materials. Sulfur is also a low cost, abundant, and environmentally friendly natural resource [8], making it a very promising cathode material candidate, especially for large scale energy storage applications [9]. However, Li/S batteries suffer from inefficient utilization of cathode materials and poor cyclability [9,10], both essentially due to the insulating nature of S and the solubility of polysulfides in liquid organic electrolytes [11,12,13,14].

Successful operation of Li/S batteries has been achieved through the development of composites of sulfur with carbonaceous [5,15,16,17,18,19,20,21,22,23] and polymeric [24,25,26,27] materials. In these composites, the S particles are embedded into the conductive carbon or polymer matrices [5,21,28], which enhance the electronic conductivity of the composite and hinder the dissolution of polysulfides into the electrolyte [13,14,28,29,30]. 

Another strategy to improve the capacity and cyclability of Li/S batteries is the electrolyte optimization so as to reduce the loss of sulfur by dissolution in the liquid electrolyte [14,31,32,33,34,35]. Among the possible electrolyte modifications, replacement of the common liquid organic electrolytes with polymer electrolytes has proved promising and efficient. 

Polymer electrolyte may generally be defined as a membrane that possesses transport properties comparable to that of common liquid ionic solutions [36]. Although the study of polymer electrolyte was started in 1973 by Fenton *et al.* [37], its technological importance was appreciated in the early 1980s [38]. Since then, a large number of polymer electrolyte systems have been prepared and characterized. It is possible and convenient to group all the polymer systems into two broad categories, *i.e.*, pure solid polymer electrolyte (SPE) and plasticized or gel polymer electrolyte systems (GPE).

The first category, pure solid polymer electrolyte, is composed of lithium salts (e.g., LiClO_4_, LiBF_4_, LiPF_6_, LiAsF_6_, LiCF_3_SO_3_, LiN(CF_3_SO_2_)_2_, LiC(CF_3_SO_2_)_3_) dissolved in high molecular weight polyether hosts, (e.g., PEO and PPO) which acts as solid solvents [39]. The ionic conduction mechanism of SPE is intimately associated with the local segmental motions of the polymer. The second category of polymer electrolyte, gel polymer electrolyte, is characterized by a higher ambient ionic conductivity but poorer mechanical properties when compared with SPE. GPE is usually obtained by incorporating a larger quantity of liquid electrolyte to a polymer matrix that is capable of forming a stable gel polymer host structure. 

Polymer electrolytes have several obvious advantages over their liquid electrolyte. Among the advantages of these electrolytes, they include no internal shorting, leakage of electrolytes and no non-combustible reaction products at the electrode surface existing in the liquid electrolytes [40,41,42,43,44]. The pre-requisites for a polymer electrolyte for lithium batteries are: high ionic conductivity at ambient and subambient temperatures, good mechanical strength, appreciable transference number, thermal and electrochemical stabilities, and better compatibility with electrodes [41,42,43,45]. In particular, for Li/S battery, it is expected that the polymer membrane can act as a physical barrier, which can help control the dissolution of the sulfide anions from the cathode and also prevent the attack of the same anions at the anode [46].

Herein, this article does not intend to review the modification of cathodes or liquid electrolytes, but it focuses on the applications of polymer electrolytes in Li/S batteries. In this review, the preparation and electrochemical properties of polymer electrolytes are studied based on the catalogue of polymer electrolytes. The electrochemical characteristics of the Li/S batteries based on these polymer electrolytes, related to the performance of their cells, are also discussed here.

## 2. Dry Solid Polymer Electrolytes in Li/S Batteries

In dry solid polymer electrolytes, the polymer host itself is used as a solid solvent along with lithium salt and it does not contain any organic liquids. As a polymer host, the high molecular weight poly(ethylene oxide) (PEO)-based solid polymer electrolytes are emerging as the best candidates to be used because of their solvation power, complexation ability and ion transport mechanism directly connected with the alkaline salt (Li^+^). However, the ionic conductivity of PEO-lithium salts (LiX) electrolytes at ambient temperature (10^−7^–10^−6^ S·cm^−1^) is not high enough for most practical applications. In order to overcome this problem, consistent research efforts have been devoted to improve the ionic conductivity of PEO-LiX (X = ClO_4_^−^, CF_3_SO_3_^−^, BF_4_^−^, PF_6_^−^, *etc.*) solid polymer electrolytes [42,47].

**Figure 1:**
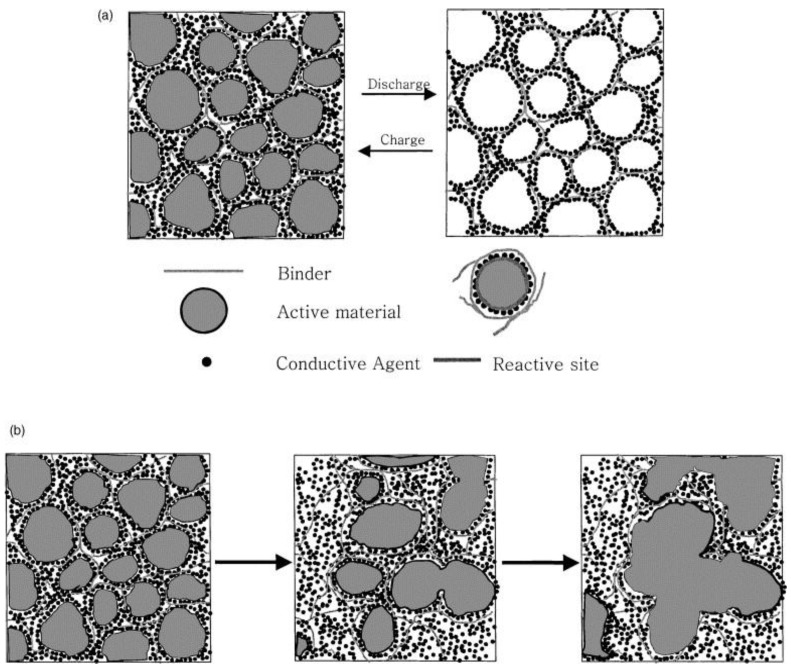
Model for morphology change of composite cathode during charge-discharge:(**a**) ideal case;(**b**) real case [48].

In Jeon *et al.*’s study [48], LiClO_4_ was chosen to dissolve in high molecular weight polymer host-PEO which acted as solid solvents. Dry polymer electrolyte made of PEO with tetra(ethylene glycol dimethyl ether) was employed into Li/S cells to study issues such as the fading capacity and low sulfur utilization. According to the change in morphologies for a composite sulfur cathode, which was obtained by scanning electron microscopy (SEM), a model for the change in morphology of the composite cathode was built as shown in Figure 1. The authors offered us a mechanism for the capacity fading was mainly due to the heterogeneity and worsening distribution of sulfur along with cycling.

Some researchers have been trying to further improve the conductivity by the use of inorganic ceramic filler such as Al_2_O_3_, SiO_2_, TiO_2_ and ZrO_2_ in the host polymer matrix [49,50,51,52,53,54,55,56,57].

In Shin *et al.*’s study [58], (PEO)_10_LiCF_3_SO_3_ polymer electrolyte with titanium oxide (Ti*_n_*O_2*n*−1_, *n* = 1, 2) was introduced into Li/S system, and they not only investigated the ionic conductivity and interfacial stability of this dry polymer electrolyte but also the discharge characteristics of Li/S cells with (PEO)_10_LiCF_3_SO_3_ polymer electrolyte. From the results of this study, titanium oxide is a good candidate as ceramic filler in (PEO)_10_LiCF_3_SO_3_ dry polymer electrolyte. Titanium Oxide filler has a size of sub-micron and several micron consisting of various phases that were prepared by ball milling for 100 h, which wereintroduced into the (PEO)_10_LiCF_3_SO_3_ polymer electrolyte. The addition of titanium oxide containing Ti_2_O_3_, TiO and Ti_2_O into the (PEO)_10_LiCF_3_SO_3_ polymer electrolyte improved the ionic conductivity due to the change of –C–O–C– vibration and ionic structure of polymer electrolyte by the decrease in crystallinity of PEO polymer electrolyte, and the interface resistance between polymer electrolyte and lithium electrode was remarkably decreased by lowering the contact area between lithium and electrolyte. 

In Jeong *et al.*’s study [59], (PEO)_6_LiBF_4_ polymer electrolyte was prepared under three different mixing conditions: stirred polymer electrolyte, ball-milled polymer electrolyte and ball-milled polymer electrolyte with 10 wt % Al_2_O_3_. The Li/S cell containing ball-milled (PEO)_6_LiBF_4_-Al_2_O_3_ polymer electrolyte delivered a high initial discharge of 1670 mAh·g^−1^, which was better than the cells with stirred (PEO)_6_LiBF_4_ polymer electrolyte or (PEO)_6_LiBF_4_ ball-milled polymer electrolyte. And also the cycle performance of Li/(PEO)_6_LiBF_4_/S cell was also remarkably improved with the addition of Al_2_O_3_.

(PEO)_20_Li(CF_3_SO_2_)_2_N-γLiAlO_2_ was prepared and introduced into Li/S battery in Wen *et al.*’s study [60]. The all-solid-state Li/S cell with PEO based polymer electrolyte operating at 75 °C exhibited an average capacity of 290 mAh·g^−1^ in 50 cycles. The cycle-stability of Li/S polymer battery was improved by amending the method to prepare the sulfur composite cathode by blending sulfur and PEO by thermal melting at 180 °C in a sealed container. The SEM results confirmed the mechanism of capacity fading [48], which suggested that the capacity of Li/S polymer battery was mostly suffered from aggregation of sulfur or lithium sulfide during cycling.

This research group did a further study to combine (PEO)_18_Li(CF_3_SO_2_)_2_N-SiO_2_ polymer electrolyte and sulfur/mesoporous-carbon composite cathode as an all solid state polymer battery [29]. The conductivity of the PEO based electrolyte could reach 5 × 10^−4^ S·cm^−1^ at 70 °C. In the sulfur cathode, mesoporous carbon sphere with the uniform channels was employed as the conductive agent, and sulfur was penetrated into those channels by a co-heating method. By this, the prepared all solid state polymer battery showed excellent cycling performance with a reversible discharge capacity of about 800 mAh·g^−1^ at 70 °C after 25 cycles.

In summary, the reason for the choosing PEO as the polymer host is mainly due to that PEO usually form stable dry complexes exhibiting a relatively higher ionic conductivity than other solvating polymers [61]. The sequential oxyethylene group: –CH_2_–CH_2_–O–, and the polar groups: –O–, –H–, –C–H–, in the polymer chains can well dissolve the ionic salts [43,44,62]. In the further study, new polymer electrolyte structures, based on the modified PEO main polymer chain with grafted polymers, block copolymers, cross-linked polymer networks, which resulted in polymer electrolytes with a lower degree of crystallinity and a low glass transition temperature *T*_g_, can be considered to employed into Li/S battery system.

## 3. Gel Polymer Electrolytes in Li/S Batteries

In the point of view of dry solid polymer, the main obstacle is still the ionic conductivity, which is generally below 10^−3^ S·cm^−1^ and not enough for practical application. At room temperature, the all solid state Li/S batteries usually showed poor performance. As a result, gel polymer electrolytes were developed [63,64,65], which can be regarded as an intermediate state between typical liquid electrolytes and dry solid polymer electrolytes. In gel polymer electrolytes, the liquid component is trapped in the polymer matrix, thereby preventing leakage of liquid electrolyte. Therefore, the pore structure of the polymer membrane is the key component and is especially important for the ionic conductivity. In Li/S battery, to date, several types of polymer membranes have been developed and characterized, such as those based on poly(ethylene oxide) (PEO), poly(vinylidene fluoride) (PVDF) and poly(vinylidene fluoride)-hexafluoropropylene (PVDF-HFP).

### 3.1. PEO-Based Gel Polymer Electrolyte

**Figure 2:**
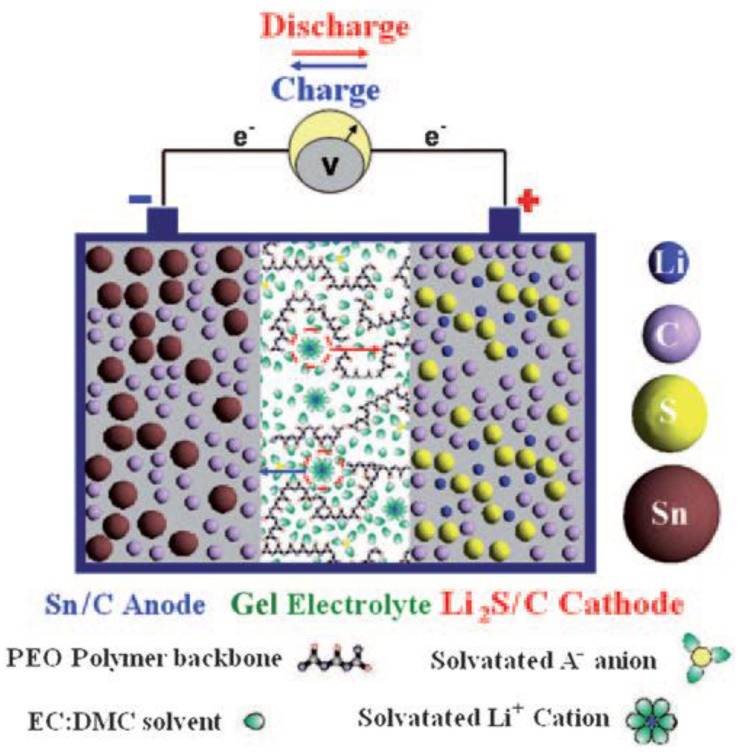
Sketch of the Sn/C/CGPE/ Li_2_S/C polymer battery developed herein. The battery is formed by a Sn/C composite anode, a PEO based gel polymer electrolyte, and a Li_2_S/C cathode. PEO = poly(ethylene oxide) [66].

**Figure 3:**
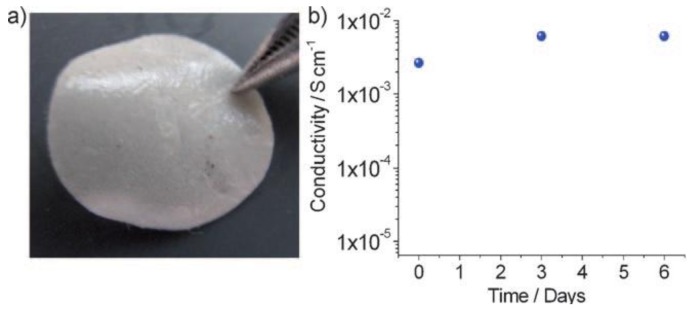
Characteristics of the PEO based gel polymer membrane to be used as electrolyte separator in the lithium-sulfur battery: (**a**) Appearance of the membrane; (**b**) Time evolution of the conductivity at room temperature [66].

Important progress was recently made by Scrosati and co-workers [66], who built a lithium metal-free new battery version as Figure 2 shows. They also renewed the electrolyte component by replacing the common liquid organic solutions with a gel-type polymer membrane, formed by trapping ethylene carbonate/dimethylcarbonate lithium hexafluorophosphate (EC: DMC/LiPF_6_) solution saturated with lithium sulfide in a polyethylene oxide/lithium trifluoromethanesulfonate (PEO/LiCF_3_SO_3_) polymer matrix [67]. A dispersed zirconia ceramic filler enhanced the mechanical properties of the gel and improved liquid retention within its bulk [68]. Impedance studies [69] indicate that the resistance of the as-prepared GPE is low and stable with time with a high conductivity approaching 10^−2^ S·cm^−1^ (Figure 3). With the assembly of the Sn/C anode, Li_2_S/C cathode and PEO based GPE, this polymer battery showed a high initial discharge of about 1200 mAh·g^−1^ at 38 mA·cm^−2^·g^−1^ (capacity calculated based on Li_2_S mass only).

### 3.2. PVDF-Based Gel Polymer Electrolyte

Poly(vinylidene fluoride) (PVDF) has received great attention as a membrane material with regard to its outstanding properties such as high mechanical strength, thermal stability, chemical resistance, and high hydrophobicity [70]. By virtue of its various appealing properties, PVDF has been chosen as a suitable polymer host. PVDF-based polymer electrolytes are expected to be highly anodically stable due to the strongly electron-withdrawing functional group (–C–F). Furthermore, PVDF itself has a high dielectric constant (ε = 8.4) for a polymer, which can assist in greater ionization of lithium salts, and thus provide a high concentration of charge carriers [39,71].

**Figure 4:**
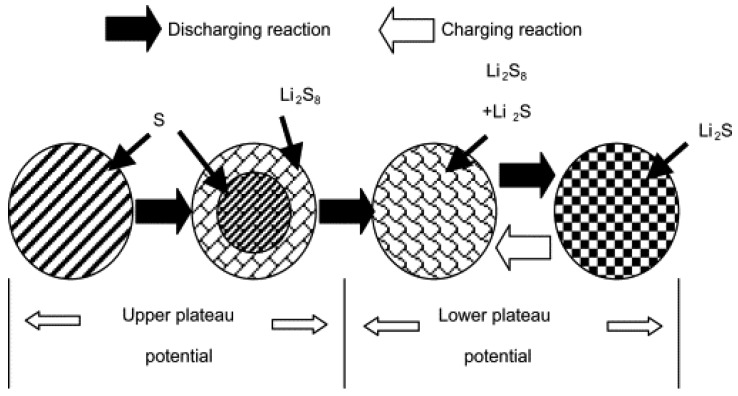
The discharge and charge reaction model of lithium/sulfur cell [72].

A detailed discussion regarding the discharge process of Li/PVDF/S was presented by Ryu *et al.* [72]. The PVDF gel polymer electrolyte was prepared by LiCF_3_SO_3_ as lithium-ion resource, tetraglyme as plasticizer, and PVDF as a gelling agent in THF solvent in Ar atmosphere. A freestanding PVDF electrolyte film was obtained after the solvent was evaporated at room temperature. By using PVDF polymer electrolyte, the Li/S cell had two plateaus-like potential regions and a discharge capacity of 1268 mAh·g^−1^ at the first discharge. The discharge capacity decreased to 1028 mAh·g^−1^ and the upper plateau region disappeared after second discharge. From XRD and DSC results of the sulfur electrode, a model was built as shown in Figure 4 to suggest that elemental sulfur disappeared and changed into Li_2_S_n_ (*n* > 4) at the upper plateau region and Li_2_S was formed at the low plateau region. 

### 3.3. PVDF-HFP Based Gel Polymer Electrolyte

Poly(vinylidene fluoride)-hexafluoropropylene (PVDF-HFP) has drawn the attention of many researchers due to its appealing properties. The high dielectric constant of ε = 8.4 facilitates for higher concentration of charge carriers, and it also comprises of both amorphous and crystalline phase; the amorphous phase of the polymer helps for higher ionic conduction, whereas the crystalline phase acts as a mechanical support for the polymer electrolyte [73,74,75].

Shin *et al.* [76] reported the preparation and performance of PVDF-HFP gel electrolyte in Li/S batteries. The PVDF-HFP gel polymer electrolyte with tetra ethylene glycol dimethylether (TEGDME) as a plasticizer, LiCF_3_SO_3_, LiBF_4_ and LiPF_6_ as lithium salt and acetone as solvent was prepared by solvent casting of slurry that mixed PVDF-HFP copolymer with acetone and salt using a ball-milling technique. This polymer electrolyte showed high mechanical property and good ionic conductivity (4.99 × 10^−4^ S·cm^−1^ at room temperature). As ball-milled gel polymer electrolytes were introduced into Li/S cells with sulfur as cathode and lithium as the anode. The first specific discharge capacities with discharge rate of 0.14 mA·cm^−2^ at room temperature were about 575 and 765 mAh·g^−1^. The melting temperature of crystalline PVDF-HFP was found to decrease, which may be due to the decrease of crystallinity by scission of the polymer chain during ball milling. Therefore, it was concluded that the ball-milling technique could be a very promising preparative technique for the preparation of slurry for polymer electrolytes.

In Wang et al’s study [15,24], a gel polymer electrolyte was formed by trapping a liquid electrolyte of PC-EC-DEC (1:4:5 v/v) containing 1 M LiPF_6_ in a dry PVDF-HFP/SiO_2_ polymer matrix. And this dry PVDF-HFP/SiO_2_ film with abundant pore structure was prepared by phase separation method. The ionic conductivity of resulting gel polymer electrolyte was about 1.2 × 10^−3^ S·cm^−1^ at room temperature. This gel polymer electrolyte was introduced into the cells with sulfur/active carbon composite cathode and sulfur/polyacrylonitrile (S/PAN) composite, respectively. The cell with S/PAN composite cathode exhibited a specific capacity up to 850 mA·g^−1^ in the initial and remained above 600 mAh·g^−1^ after 50 cycles. With elemental sulfur incorporated in porous carbon, S/C composite exhibited reversible capacity of 440 mAh·g^−1^ at current density of 0.3 mA·cm^−2^.

In summary, gel polymer electrolyte was considered as liquid electrolyte trapped in the polymer membrane. When the bulk of the membrane was composed of connected micropores, the ion conductivity of the gel polymer electrolyte mainly depended on the property of the liquid electrolyte. Otherwise, if the prepared membrane did not have many connected pores, the transfer of Li^+^ mainly happens in the polymer membrane [77]. Therefore, in the further study, modifying the pore structure of the membrane and changing the crystallinity of the polymer matrix were developed as the most important strategies to improve Li^+^ transport and ion conductivity of GPE in Li/S battery . The former was mainly achieved by optimizing the preparation methods and the latter by modifications of polymer matrix, such as blending, copolymer and cross-linking, compounding, and adding nanofillers [77].

## 4. Conclusions

Li/S batteries provide much hope, but also many challenges. In general, the main problem in an Li/S battery is its poor cyclic ability, which is mainly caused by polysulfides dissolving into the electrolyte. To solve this problem, the polymer electrolyte is introduced into Li/S battery. As discussed above, with employment of a dry polymer electrolyte and gel polymer electrolyte, the cell showed a better cycle performance. However, problems in Li/S batteries, such as aggregation of sulfur or lithium sulfide during cycling, could not be solved merely by modifying the electrolyte. Together with advances in anodes and cathodes, the development of polymer electrolytes with high conductivity, high compatibility and mechanical strength, can offer a promising future for Li/S batteries.

## References

[1] Song D., Ikuta H., Uchida T., Wakihara M. (1999). The spinel phases LiAl_*y*_Mn_2−*y*_O_4_ (*y* = 0, 1/12, 1/9, 1/6, 1/3) and Li(Al,M)_1/6_Mn_11/6_O_4_ (M = Cr, Co) as the cathode for rechargeable lithium batteries. Solid State Ionics.

[2] Bakenov Z., Taniguchi I. (2005). Electrochemical performance of nanostructured LiM_x_Mn_2−x_O_4_ (M = Coand Al) powders at highcharge-discharge operations. Solid State Ionics.

[3] Kang B., Ceder G. (2009). Battery materials for ultrafast charging and discharging. Nature.

[4] Whittingham M.S. (2004). Lithium batteries and cathode materials. Chem. Rev..

[5] Ji X.L., Lee K.T., Nazar L.F. (2009). A highly ordered nanostructured carbon-sulphur cathode forlithium-sulphur batteries. Nat. Mater..

[6] Sun Y.K., Myung S.T., Park B.C., Prakash J., Belharouak I., Amine K. (2009). High-energy cathode material for long-life and safe lithium batteries. Nat. Mater..

[7] Yang Y., McDowell M.T., Jackson A., Cha J.J., Hong S.S., Cui Y. (2010). New nanostructured Li_2_S/silicon rechargeable battery with high specific energy. Nano Lett..

[8] Tischer R.P. (1983). The Sulfur Electrode.

[9] Marmorstein D., Yu T.H., Striebel K.A., McLarnon F.R., Hou J., Cairns E.J. (2000). Electrochemical performance of lithium/sulfur cells with three different polymer electrolytes. J. Power Sources.

[10] Yamin H., Peled E. (1983). Electrochemistry of a nonaqueous lithium/sulfur cell. J. Power Sources.

[11] Rauh R.D., Abraham K.M., Pearson G.F., Surprenant J.K., Brummer S.B. (1979). A lithium/dissolved sulfur battery with an organic electrolyte. J. Electrochem. Soc..

[12] Yamin H., Gorenshtein A., Penciner J., Sternberg Y., Peled E. (1988). Lithium sulfur battery. J. Electrochem. Soc..

[13] Shim J., Striebel K.A., Cairns E.J. (2002). The lithium/sulfur rechargeable cell. J. Electrochem. Soc..

[14] Cheon S.E., Ko K.S., Cho J.H., Kim S.W., Chin E.Y., Kim H.T. (2003). Rechargeable lithium sulfur battery. J. Electrochem. Soc..

[15] Wang J.L., Yang J., Xie J.Y., Xu N.X., Li Y. (2002). Sulfur-carbon nano-composite as cathode for rechargeable lithium battery based on gel electrolyte. Electrochem. Commun..

[16] Han S.C., Song M.S., Lee H., Kim H.S., Ahn H.J., Lee J.Y. (2003). Effect of multiwalled carbon nanotubes on electrochemical properties of lithium/sulfur rechargeable batteries. J. Electrochem. Soc..

[17] Zheng W., Liu Y.W., Hu X.G., Zhang C.F. (2006). Novel Nanosized Adsorbing Sulfur Composite Cathode Materials for the Advanced Secondary Lithium Batteries. Electrochim. Acta.

[18] Yuan L.X., Yuan H.P., Qiu X.P., Chen L.Q., Zhu W.T. (2009). Improvement of cycle property of sulfur-coated multi-walled carbon nanotubes composite cathode for lithium/sulfur batteries. J. Power Sources.

[19] Chen S.R., Zhai Y.P., Xu G.L., Jiang Y.X., Zhao D.Y., Li J.T., Huang L., Sun S.G. (2011). Ordered mesoporous carbon/sulfur nanocomposite of high performances as cathode for lithium-sulfur battery. Electrochem. Acta.

[20] Wang J., Chew S.Y., Zhao Z.W., Ashraf S., Wexler D., Chen J., Ng S.H., Chou S.L., Liu H.K. (2008). Sulfur-mesoporous carbon composites in conjunction with a novel ionic liquid electrolyte for lithium rechargeable batteries. Carbon.

[21] Zhang B., Qin X., Li G.R., Gao X.P. (2010). Enhancement of long stability of sulfur cathode by encapsulating sulfur into micropores of carbon spheres. Energy Environ. Sci..

[22] Zhang B., Lai C., Zhou Z., Gao X.P. (2009). Preparation and electrochemical properties of sulfur-acetylene black composites as cathode materials. Electrochim. Acta.

[23] Wang H.L., Yang Y., Liang Y.Y., Robinson J.T., Li Y.G., Jackson A., Cui Y., Dai H.J. (2011). Graphene-wrapped sulfur particles as a rechargeable lithium-sulfur battery cathode material with high capacity and cycling stability. Nano Lett..

[24] Wang J.L., Yang J., Wan C.R., Du K., Xie J.Y., Xu N.X. (2003). Sulfur composite cathode materials for rechargeable Lithium batteries. Adv. Funct. Mater..

[25] Wang J., Chen J., Konstantinov K., Zhao L., Ng S.H., Wang G.X., Guo Z.P., Liu H.K. (2006). Sulfur-polypyrrole composite positive electrode materials for rechargeable lithium batteries. Electrochim. Acta.

[26] Sun M.M., Zhang S.C., Jiang T., Zhang L., Yu J.H. (2008). Nano-wire networks of sulfurpolypyrrole composite cathode materials for rechargeable lithium batteries. Electrochem. Commun.

[27] Liang X., Liu Y., Wen Z.Y., Huang L.Z., Wang X.Y., Zhang H. (2011). A nano-structured and highly ordered polypyrrole-sulfur cathode for lithium-sulfur batteries. J. Power Sources.

[28] Zhang Y.G., Bakenov Z., Zhao Y., Konarov A., Doan T.N.L., Malik M., Paron T., Chen P. (2012). One-step synthesis of branched sulfur/polypyrrole nanocomposite cathode for lithium rechargeable batteries. J. Power Sources.

[29] Liang X., Wen Z., Liu Y., Zhang H., Huang L., Jin J. (2011). Highly dispersed sulfur in ordered mesoporous carbon sphere as a composite cathode for rechargeable polymer Li/S battery. J. Power Sources.

[30] Mikhaylik Y.V., Akridge J.R. (2004). Polysulfide shuttle study in the Li/S battery system. J. Electrochem. Soc..

[31] Liang X., Wen Z., Liu Y., Wu M., Jin J., Zhang H., Wu X. (2011). Improved cycling performances of lithium sulfur batteries with LiNO_3_^−^ modified electrolyte. J. Power Sources.

[32] Choi J.W., Kim J.K., Cheruvally G., Ahn J.H., Ahn H.J., Kim K.W. (2007). Rechargeable lithium/sulfur battery with suitable mixed liquid electrolytes. Electrochimica. Acta.

[33] Chang D.R., Lee S.H., Kim S.W., Kim H.T. (2002). Binary electrolyte based on tetra(ethylene glycol) dimethyl ether and 1,3-dioxolane for lithium-sulfur battery. J. Power Sources.

[34] Kim S., Jung Y., Park S.J. (2005). Effects of imidazolium salts on discharge performance of rechargeable lithium-sulfur cells containing organic solvent electrolytes. J. Power Sources.

[35] Choi J.W., Cheruvally G., Kim D.S., Ahn J.H., Kim K.W., Ahn H.J. (2008). Rechargeable lithium/sulfur battery with liquid electrolytes containing toluene as additive. J. Power Sources.

[36] Stephan A.M. (2006). Review on gel polymer electrolytes for lithium batteries. Eur. Polym. J..

[37] Fenton D.E., Parker J.M., Wright P.V. (1973). Complexes of alkali metal ions with poly(ethylene oxide). Polymer.

[38] Shriver D.F., Bruce P.G. (1995). Solid State Electrochemistry.

[39] Song J.Y., Wang Y.Y., Wan C.C. (1999). Review of gel-type polymer electrolytes for lithium-ion batteries. J. Power Sources.

[40] Gray F.M. (1991). Solid Polymer Electrolytes-Fundamentals and Technological Applications.

[41] Scrosati B. (1993). Applications of Electroactive Polymers.

[42] Gray F.M. (1997). Polymer Electrolytes.

[43] MacCallum J.R., Vincent C.A. (1987). Polymer Electrolytes Reviews-I.

[44] MacCallum J.R., Vincent C.A. (1989). Polymer Electrolytes Reviews-II.

[45] Idris N.H., Rahman M.M., Wang J.Z., Liu H.K. (2012). Microporous gel polymer electrolytes for lithium rechargeable battery application. J. Power Sources.

[46] Kim K.M., Park N.G., Ryu K.S., Chang S.H. (2006). Characteristics of PVdF-HFP/TiO_2_ composite membrane electrolytes prepared by phase inversion and conventional casting methods. Electrochim. Acta.

[47] Weston J.E., Steele B.C.H. (1982). Effects of inert fillers on the mechanical and electrochemical properties of lithium salt-poly(ethylene oxide) polymer electrolytes. Solid State Ionics.

[48] Jeon B.H., Yeon J.H., Kim K.M., Chung I.J. (2002). Preparation and electrochemical properties of lithium-sulfur polymer batteries. J. Power Sources.

[49] Croce F., Persi L., Scrosati B., Serraino-Fiory F., Plichta E., Hendrickson M.A. (2001). Role of the ceramic fillers in enhancing the transport properties of composite polymer electrolytes. Electrochim. Acta.

[50] Dissanayake M.A.K.L., Jayathilake P.A.R.D., Bokalawela R.S.P., Albinsson I., Mellander B.E. (2003). Effect of concentration and grain size of alumina filler on the ionic conductivity enhancement of the (PEO)_9_LiCF_3_SO_3_:Al_2_O_3_ composite polymer electrolyte. J. Power Sources.

[51] Ahn J.H., Wang G.X., Liu H.K., Dou S.X. (2003). Nanoparticle-dispersed PEO polymer electrolytes for Li batteries. J. Power Sources.

[52] Appetecchi G.B., Croce F., Persi L., Ronci F., Scrosati B. (2000). Transport and interfacial properties of composite polymer electrolyte. Electrochim. Acta.

[53] Jayathilake P.A.R.D., Dissanayake M.A.K.L., Albinsson I., Mellander B.E. (2002). Effect of nano-porous Al_2_O_3_ on thermal, dielectric and transport properties of the (PEO)_9_LiTFSI polymer electrolyte system. Electrochim. Acta.

[54] Lin C.W., Hung C.L., Venkateswarlu M., Hwang B.J. (2005). Influence of TiO_2_ nano-particles on the transport properties of composite polymer electrolyte for lithium-ion batteries. J. Power Sources.

[55] Xi J., Qiu X., Ma X., Cui M., Yang J., Tang X., Zhu W., Chen L. (2005). Composite polymer electrolyte doped with mesoporous silica SBA-15 for lithium polymer battery. Solid State Ionics.

[56] Chung S.H., Wang Y., Persi L., Croce F., Greenbaum S.G., Scrosati B., Plichta E. (2001). Enhancement of ion transport in polymer electrolytes by addition of nanoscale inorganic oxides. J. Power Sources.

[57] Croce F., Settimi L., Scrosati B. (2006). Superacid ZrO_2_-added, composite polymer electrolytes with improved transport properties. Electrochem. Commun..

[58] Shin J.H., Kim K.W., Ahn H.J., Ahn J.H. (2002). Electrochemical properties and interfacial stability of (PEO)_10_LiCF_3_SO_3_-Ti_*n*_O_2*n*-1_ composite polymer electrolytes for lithium/sulfur battery. Mater. Sci. Eng. B.

[59] Jeong S.S., Lim Y.T., Choi Y.J., Cho G.B., Kim K.W., Ahn H.J., Cho K.K. (2007). Electrochemical properties of lithium sulfur cells using PEO polymer electrolytes prepared under three different mixing conditions. J. Power Sources.

[60] Zhu X., Wen Z., Gu Z., Lin Z. (2005). Electrochemical characterization and performance improvement of lithium/sulfur polymer batteries. J. Power Sources.

[61] Agrawa R.C., Pandey G.P. (2008). Solid polymer electrolytes: Materialdesigning and all-solid-state batteryapplications: An overview. J. Phys. D Appl. Phys..

[62] Gray F.M. (1991). Polymer Electrolytes: Fundamentals and Technological Applications.

[63] Wu Y.P., Zhang H.P., Wu F., Li Z.H. (2007). Polymer Lithium-Ion Batteries.

[64] Xu J.J., Ye H. (2005). Polymer gel electrolytes based on oligomeric polyether/cross-linked PMMA blends prepared via in situ polymerization. Electrochem. Commun..

[65] Oliver M. (1997). Blended Polymer Gel Electrolytes. U.S. Patent.

[66] Hassoun J., Scrosati B. (2010). A high-performance polymer tin sulfur lithium ion battery. Angew. Chem. Int. Ed..

[67] Scrosati B., Scrosati S. (2002). Lithium polymer electrolytes. Advances in Lithium Ion Batteries.

[68] Appetecchi G.B., Romagnoli P., Scrosati B. (2001). Composite gel membranes: A new class of improved polymer electrolytes for lithium batteries. Electrochem. Commun..

[69] MacDonald J.R., Barsoukov E. (2005). Impedance Spectroscopy, Theory, Experiment and Applications.

[70] Liu F., Hashim N.A., Liu Y., Abed M.R.M., Li K. (2011). Progress in the production and modification of PVDF membranes. J. Membrane Sci..

[71] Watanabe M., Kanba M., Matsuda H., Mizoguchi K., Shinohara I., Tsuchida E., Tsunemi K. (1981). High lithium ionic conductivity of polymeric solid electrolytes. Makromol. Chem Rapid Commun..

[72] Ryu H.S., Ahn H.J., Kim K.W., Ahn J.H., Lee J.Y. (2006). Discharge process of Li/PVdF/S cells at room temperature. J. Power Sources.

[73] Stephan A.M., Nahm K.S., Kulandainathan M.A., Ravi G., Wilson J. (2006). Poly(vinylidene fluoride-hexafluoropropylene) (PVdF-HFP) based composite electrolytes for lithium batteries. Eur. Polym. J..

[74] Stephan M.A., Dale T. (2003). Charge-discharge studies on a lithium cell composed of PVdF-HFP polymer membranes prepared by phase inversion technique with a nanocomposite cathode. J. Power Sources.

[75] Stephan M.A., Dale T. (2003). Characterization of PVdF-HFP polymer membranes prepared by phase inversion technique I: Morphology and charge discharge studies. Electrochim. Acta.

[76] Shin J.H., Jung S.S., Kim K.W., Ahn H.J. (2002). Preparation and characterization of plasticized polymer electrolytes based on the PVdF-HFP copolymer for lithium/sulfur battery. J. Mater. Sci. Mater. Electron..

[77] Li G.C., Li Z.H., Zhang P., Zhang H.P., Wu Y.P. (2008). Research on a gel polymer electrolyte for Li-ionbatteries. Pure Appl. Chem..

